# Spectrum of Intracerebral Hemorrhage in Children: A Report from PICU of a Resource Limited Country

**DOI:** 10.1155/2016/9124245

**Published:** 2016-01-03

**Authors:** Qalab Abbas, Qurat ul Ain Merchant, Bushra Nasir, Anwar ul Haque, Basit Salam, Gohar Javed

**Affiliations:** ^1^Department of Pediatrics and Child Health, Aga Khan University, Karachi, Pakistan; ^2^Department of Radiology, Aga Khan University, Karachi, Pakistan; ^3^Department of Surgery, Section of Neurosurgery, Aga Khan University, Karachi, Pakistan

## Abstract

Intracerebral hemorrhage (ICH) in children is a rare but disabling disease that accounts for almost half cases of stroke. We report our experience of ICH in children. Retrospective review of medical records of children (1 month-16 years) admitted in Pediatric Intensive Care Unit between January 2007 and December 2014 was done. Data collected included age, gender, presentation, examination findings, neuroimaging done (CT, MRI, and angiography) management (conservative/intervention), and outcome. Results are presented as frequency and percentages. Of the total 50 patients, 58% were male and 26% were <1 year. On presentation 44% had vomiting, 42% had seizures, and GCS < 8 while 40% had altered level of consciousness. Single bleed was present in 88%, 94% had supratentorial bleed, and 32% had intraventricular extension. 72% had bleed volume of <30 mL and 8% had >60 mL. CT scan was done in 98% patients and MRI in 34%, while 6% underwent conventional angiography. 60% patients were managed conservatively, 36% underwent neurosurgical intervention, and 6% underwent radiological vascular intervention. Hematologic causes were identified in 52% patients and vascular malformations in 14% and in 26% no cause could be identified. 26% of patients expired.

## 1. Introduction

Intracerebral hemorrhage (ICH) in children is a rare but often disabling disease, leading to high rates of morbidity and mortality in this population [1]. It accounts for almost fifty percent of all the cases of stroke in children [2, 3]. Various pediatric studies on ICH report different values depending on differences in the population studied and difference in the sensitivity and timing of diagnostic modalities used. Owing to the rarity of this entity and a potentially fatal course, it poses a diagnostic as well as therapeutic challenge to physicians in the current era [1].

The most common causes of spontaneous ICH in children are found to be vascular malformations like arteriovenous malformations (AVM), aneurysms, and cavernous angioma [4]. These account for almost 40% to 57% of cases of spontaneous ICH. Other causes include bleeding disorders: acquired and congenital. Hematological disorders are reported to be the major risk factor in 10% to 30% of the hemorrhagic strokes in most studies. Most cases of acquired coagulopathies result from aplastic anemia, idiopathic thrombocytopenic purpura (ITP), neoplasia, and thrombocytopenia of chronic kidney and liver disease. Congenital disorders include hemophilia A, hemophilia B, and rarer diseases like von Willebrand factors (vWF) deficiency and FXIII deficiency among others [4]. Despite the advent of newer coagulation assays and neuroimaging techniques, many cases of brain hemorrhage in children remain unexplained.

Much remains to be known about its epidemiology from our part of the world as it is relatively less common disease and data is limited to mainly case series [5–7]. We report our experience of the clinical presentation, etiology, and outcome of ICH in children at our Pediatric Intensive Care Unit (PICU).

## 2. Methods

A retrospective review of medical records of children (age of 1 month to 18 years), admitted between 2007 and 2014 in our PICU with spontaneous ICH, was performed. We selected this year duration because of more frequent use of CT and MRI during this era. Our PICU is a multidisciplinary-cardiothoracic closed ICU with average of 450 annual admissions and 24-hour coverage by a senior resident and is supervised by a trained (American Board Certified) pediatric intensivist. There are no dedicated pediatric neurosurgeons in our hospital. A dedicated radiological suit is present with round-the-clock service for all the diagnostic testing and procedure including CT scan, MRI, MRA, and interventional radiology.

A total of 50 patients were identified. Data collected included demographics (age and sex), etiology, clinical presentation, neuroimaging investigations, and neurological examination at admission, anatomic location, surgical procedures, therapy, and immediate outcome (survival versus death) on a structured pro forma. The diagnosis was confirmed by CT, MRI, and different hematological investigations as required. Radiographic studies were reviewed with the radiologist whenever possible. The volume of intracerebral hematoma was calculated on CT scan images in all the patients except one (on MRI) according to ABC/XYZ volume estimation on the first imaging done [2].

Preterm infants born at less than 37 weeks' gestation and term infants with pure intraventricular hemorrhages were excluded. This study was approved by the Ethical Review Committee (ERC) of the institute (ERC number 3092-PED-ERC-14).

Analysis was done using SPSS version 19. Results are presented as frequency and percentages and mean and standard deviation (SD). Chi square test was applied for possible risk factors associated with bad outcome and *p* value of <0.05 was considered significant.

## 3. Results

Out of the 50 children admitted in our PICU with ICH, mean age of the patients was 7.1 years (SD ± 5.6), 26% of children were <1 years, and 29 (58%) children were males. The presenting symptoms included vomiting in 44% and seizures in 42%, followed by loss of consciousness in 40% and headache in 28%. Focal deficits were seen in 8% of cases. Glasgow coma scale (GCS) of <8 was present in 42%. Mean PRISM III score on presentation was 7.3 SD ± 6.3 (Table 1).

Seven cases (14%) were due to vascular malformation, 6 cases were due to AVM, and one case was due to an aneurysm. Laboratory examinations detected 4 cases of acute leukemia, 6 cases of aplastic anemia, 3 cases of ITP, 7 cases of vitamin K deficiency bleeding, and a single case of disseminated intravascular coagulation (DIC). Factor VIII and factor XIII deficiency were identified in 3 and 2 patients, respectively. One patient developed ICH after pseudomonas meningitis, and one each after acute liver failure, viral encephalitis and thalassemia. Etiology remained undetermined in 13 patients (26%).

CT was performed in 49/50 (98%) cases, and CT scan alone was done in 14 patients; course of two of these patients was too short to allow for further investigation while the rest of these patients had underlying hematological cause. CT angiography was performed in in 13% of patients, MRI was performed in in 33% of patients, and Magnetic Resonance Angiography (MRA) was performed in in 10% of patients while conventional angiography was performed in 3 patients. Single and supratentorial bleed was present in 44 (88%) and 47 (94%) patients, respectively. There were 49 (97%) patients intraparenchymal bleed, 6 (12%) patients with subarachnoid bleed, and 2 patients with subdural bleed. Eight cases (16%) had multiple episodes of ICH. Three patients with multiple episodes of ICH were suffering from acute leukemia while one child had developed DIC.

The intraparenchymal bleed was further subdivided into supratentorial and infratentorial. Of the supratentorial bleed, 4 patients had intraventricular hemorrhage, 39 (76.5%) were lobar, and 6 (11.8%) primarily involved the basal ganglia and thalamus. Of the lobar cases, 21 cases were predominantly frontal, 4 cases were parietal, and 14 cases were temporal in location. Intraventricular extension was seen in 11 cases of cerebral hemorrhages.

Only 4 (8%) patients had volume of bleed >60 mL and two of them died while 10 (20%) had 30–60 mL and 37 (74%) had <30 mL. Conservative management was done in 30 (60%) patients, while 18 (36%) patients underwent neurosurgical procedure that included evacuation of hematoma, decompressive craniotomy, or clipping of aneurysms. Three patients underwent vascular interventional radiology procedure.

The mean length of hospital stay was 8.9 days (SD ± 7.8), with a range from 0 (i.e., patients who survived <1 day) to 39 days.

The in-hospital survival rate of all patients was 74% as 13 patients expired (26%) with 9 patients fulfilling the criteria for brain death. Patients with GCS < 8 had higher mortality rates (*p* value 0.017, OR 3.21, and 95% CI 1.13–9.07) (Table 2).

## 4. Discussion

The age and gender distribution of our patients are similar to other reports (mean age of 7 years and M : F (1.3 : 1)) [7–9]. The presentation of ICH in our series was with vomiting (44%), seizures (42%), and altered consciousness (40%) and only 8% had focal neurological signs. Kumar et al. case series showed symptoms of increased intracerebral pressures (70%) and altered consciousness (50%) as the main presentation in their patients and less patients had seizures (28%) and weakness (36%) [10]. Headache was present in 28% of our patients while some series report higher number (73%) [4]. This may be due to age differences as younger children are not able to communicate headache. Almost the same results are shown by Al-Jarallah et al. [8].

A recent population based study reported a distribution of risk factors that was similar to many previous reports in that most cases of ICH were caused by structural vascular lesions like AVMs [11]. Our study showed that AVMs were the second most frequent cause for ICH (7/50 13.7%).

As a result of better management of the rare chronic etiologies of ICH (brain tumor and congenital heart disease) in children over the past decades, the etiology of ICH in children has transitioned to these chronic diseases. Hematological disorders such as aplastic anemia (5/50 10%), ITP (3/50), and acute leukemia (4/50 8%) are the most frequent group of disorders leading to ICH in our study group. Similarly, Al-Jarallah et al. also reported bleeding disorders in 32% of their patients [8]. Congenital disorders such as FVIII deficiency (3/50) and FXIII deficiency (2/50) also led to ICH in children in our series. This high incidence of ICH secondary to bleeding disorders in our study can be explained by high number of children treated for hematological disorders at our institute.

Our study reported a substantial number of ICH (7/50 13.7%) due to vitamin K deficiency bleeding (VKDB) which generally occurs in young infants [12, 13]. Although administration of intramuscular vitamin K in the postnatal period has dramatically reduced the incidence VKDB, rare sporadic cases with late onset hemorrhage have been described in exclusively breast-fed children despite this [12, 13]. Other causes include malabsorption and hepatobiliary disease. But in our case series it could be due to high number of home-deliveries in Pakistan, where IM vitamin K is not administered to the neonate.

Like of Al-Jarallah et al., the main location of hemorrhage was supratentorial [8, 9]. The most common site of supratentorial hemorrhage was found to be lobar in our series (76.5%), in concordance with Lin et al. who found lobar ICH in 50% of their cases [6].

Management strategies primarily focus on prevention of rebleeding, minimizing neurological deficits, and decreasing the risk of hematoma expansion and cerebral ischemia. Management options include supportive management in the PICU with neuroprotective strategies and surgical procedures for evacuation of the intracerebral hematoma in selected cases.

Different risk factors like volume, size, and location of hemorrhage have been described to be associated with bad outcome. Comatose state (GCS < 8), infratentorial bleeds, and age younger than 3 years are poor prognostic factors as determined by previous studies. Although our series confirms the morbidity and mortality depending on GCS, we were not able to find the significant association with other risks already identified.

In contrast to the mortality seen in children with primary lesions like AVMs (40–42%) in the literature, our study reports no deaths in patients with AVMs.

Being a single center retrospective PICU experience findings may not be the true population representation.

This is the first comprehensive report from PICU of our country where resources for advanced investigation and therapeutic modalities are still scarce.

## 5. Conclusion 

ICH still remains a disabling disease with no underlying identifiable cause in many cases. A high index of suspicion is of paramount importance in early recognition and management of children with this problem. Long-term morbidity and mortality are considerable, especially in the cases of infratentorial hemorrhage and in children with hematological disorders.

## Figures and Tables

**Table 1:** Basic demographic and clinical data (*n* = 50).

Variable	Number	Percentage
Gender (male)	29	58
Age		
<1 year	13	26
>1 year	37	74
Symptoms		
Headache	14	28
Vomiting	22	44
Altered consciousness	20	40
Seizures	21	42
Hemiparesis	4	8
GCS < 8	21	42
Single bleed	44	88
Supratentorial	47	94
Intraventricular extension	16	32
Volume		
<30 mL	36	72
30–60 mL	10	20
>60 mL	4	8
Investigations		
CT scan	49	98
CT angiography	7	14
MRI	17	34
MRA	5	10
Conventional angiography	3	6
Treatment		
Conservative	30	60
Neurosurgical	18	36
VIR^*∗*^	3	6
Etiology		
Vascular	7	14
Hematologic	26	52
Miscellaneous	4	8
Idiopathic	13	26

^*∗*^VIR = vascular interventional radiology.

**Table 2:** Comparison of different variables with outcome (*n* = 50).

Variable	Alive (*n* = 38)	Expired (*n* = 13)	*p* value and odds ratio
Age			
<1 year	11 (29)	2 (15)	0.33 OR 2.24 (0.425–11.80)
>1 year	26 (72)	11 (85)	
Gender (male)	21	8	0.36 OR 0.65 (0.18–1.24)
Headache	11	3	—
Vomiting	14	8	—
Altered consciousness	15	5	—
Seizures	17	4	—
GCS < 8	12	9	0.01 OR 3.21 (1.13–9.07)
Hemiparesis	3	1	—
Multiple bleed	3	4	0.06 OR 5.18 (0.97–27.45)
Supratentorial	33	14	0.22 OR 0.723 (0.606–0.863)
Intraventricular extension	10	6	—
Volume			
<60	35	11	0.24 OR = 3.27, (0.412–26.01)
>60	2	2	
Management			
Conservative	17	9	—
Neurosurgical	17	4	
VIR	2	1	
